# Supplementary material for: “It’s his cheerfulness that gives me hope”: A qualitative analysis of access to pediatric cancer care in Northern Tanzania
Source: PLOS Glob Public Health. 2024 Dec 11;4(12):e0003503. doi: 10.1371/journal.pgph.0003503 (PMC11633992; doi:10.1371/journal.pgph.0003503)
Supplement: Attachment — Submitted filename: PLOS_Peds Cancer_Response to Reviewers.docx [file pgph.0003503.s003.docx]

October 9, 2024

To whom it may concern,

We appreciate the helpful suggestions and questions raised and thank the reviewers for the opportunity to improve our manuscript. We have incorporated the changes requested with our specific modifications outlined below. Our revised manuscript has been attached to this resubmission packet.

**Reviewer #1:**

**Comment:** This is an interesting manuscript utilizing a model introduced for obstetrics to describe a common problem in LMICs. The manuscript gives an interesting post-mortem of the problem of delayed treatment from the standpoint of the dynamics within the healthcare system in Tanzania. The limitation of the study is the near uniformity of the participants and the fact that they were mostly low socio-economic class families. It is a well thought out and executed research endeavor.

*Response: We thank the reviewer for this thoughtful comment and critique of our manuscript.*

**Reviewer #2:** 
**Comment:** Congratulations on your work! This is an interesting and a relevant study. I added below some suggestions.

*Response: We thank the reviewer for this comment and the proceeding suggestions to improve our manuscript for publication.*

**ABSTRACT**
**Comment:** Sampling and sample size were not clearly described, e.g., purposive sampling and saturation with 13 IDIs. This should be aligned with the methods section in the main text.

*Response: We appreciate this important comment regarding our abstract. We have edited the methods section of the abstract to more clearly articulate participant sampling and guidelines for data collection and saturation. Particularly, we have included, “Using purposive sampling methods, parents and caregivers of children obtaining pediatric cancer care at KCMC were approached for participation in in-depth interviews (IDIs) and a demographic survey.” And, “Data collection concluded once saturation was achieved at 13 IDIs, defined as the absence of new codes after three consecutive interviews.” We hope these revisions more accurately introduce our methodology.*

**Comment:** In the results section, I wonder if you could list the themes clearly.

*Response: We appreciate this suggestion from the reviewer. We have revised the results section of the abstract to more clearly list themes within the delays. However, we were significantly limited by word count due to necessary additions to the methods section of the abstract. “In the first delay, themes included waiting for symptoms to resolve and the identification of initial symptoms. The most substantial delays occurred in delay 2, including health infrastructure at mid-level facilities, misdiagnoses, the referral system, travel, and traditional medicine. Participants did not describe delays after arrival to KCMC and rather offered perspective on their child’s cancer diagnosis, their concerns while obtaining care, and their hopes for the future.” We hope these revisions better describe the thematic codes.*

**INTRODUCTION**
**Comment:** A great highlight was included about the capacity of pediatric oncology services in Tanzania. Well done. I wonder if you could elaborate a bit and describe if you had other challenges like trained pediatric oncology nurses; and how the local health care system deals with the demand on the pediatric oncology services by such a large number of newly diagnosed patients annually.

*Response: We appreciate this comment from the reviewer. We have revised the introduction to describe current training initiatives for pediatric and adult oncology care in Tanzania. “Due to workforce capacity challenges, training initiatives at Muhimbili University of Health and Allied Sciences have offered specialized training for nurses in both adult and pediatric oncology care.”*

**Comment:** A minor suggestion, consider removing the linking words, like “Further”

*Response: We appreciate this suggestion and have revised the manuscript to limit the amount of linking words/phrases.*

**METHODS**
**Comment:** It is great to read about the research team and reflexivity. I wonder if you could add the team positionality.

*Response: We appreciate this important feedback from the reviewer. We have updated the “Research Team and Reflexivity” section of our manuscript to specifically acknowledge the positionality of both the first and last authors who led study design, data collection, analysis, and reporting efforts with our partners in Tanzania – “Before proceeding with the methods and findings from this study, the first and last author acknowledge their positionality as educated, White American women within an influential U.S. institution. Although actions were taken to support equitable partnership and limit bias, the authors recognize that this positionality may have inherently influenced the study design and interpretations of data.”*

**Comment:** For purposive sampling, I wonder if you could elaborate a bit and describe how you achieved the maximum variation of the sample.

*Response: We thank the reviewer for this feedback regarding our sampling strategy. We have revised the “Participant Screening and Enrollment” section to more clearly articulate our rationale and approach to purposive sampling. Specifically, “In accordance with purposive sampling principles, KCMC’s pediatric oncologist (EM) and the local research coordinator (HK) screened and enrolled participants to represent a wide range of cancer diagnoses, child ages, demographic characteristics, and experiences in obtaining cancer care.”*

**Comment:** Could you elaborate a bit and describe how the field notes were used in the analysis? How about triangulation?

*Response: We appreciate this important question. We have revised the data collection and analysis sections of our manuscript to better describe the use of field notes and triangulation of data. First, we have added, “Field notes were not directly used in the final qualitative analysis, rather they helped frame and provide context regarding the findings.” Regarding triangulation and validity of findings, we have revised the analysis section to state, “For the triangulation of data, all analysts (MM, HK, PE) compared and discussed interpretations to ensure consistency and address and discrepancies in coding. A coding comparison query resulted in >90% coder agreeability between the three coders. This collaborative approach enhanced the validity and reliability of findings by incorporating diverse perspectives, limiting individual biases, and reinforcing identified themes.”*

**DISCUSSION**
Comment: I wonder if you could link the lack of local resources (e.g., 1 oncologist for 100 to 150 patients a year, and the lack of trained pediatric oncology nurses) with the reported factors and how that would lead to delays in diagnosis/treatment access and treatment abandonment after the child start receiving treatment.

*Response: We appreciate this suggestion from the reviewer. To note, no participants suggested delays once they reached KCMC as a final treatment facility. However, we have revised the discussion to note workforce capacity challenges in smaller, rural communities – “Due to the complexities of recognizing signs of pediatric cancer and the lack of specialized oncology services in rural communities, we recommend that community health workers (CHWs) and traditional healers be trained to identify and suggest when further medical treatment should be sought.” Additionally, we have included an example and suggestion for workforce education and training collaborations at smaller health facilities. “In addition, it will be critical that the capacity to recognize children with potential cancer conditions improves at smaller health facilities. As a zonal referral hospital, KCMC has built a collaboration with Princess Máxima Center for Pediatric Oncology to provide biannual, specialized pediatric oncology education to all KCMC oncology staff. To minimize disease progression and household OOPs costs, similar collaborations or training initiatives should be considered at smaller, community health facilities.” We hope these revisions strengthen our discussion for publication.*
